# Hospital management practices in county-level hospitals in rural China and international comparison

**DOI:** 10.1186/s12913-021-07396-y

**Published:** 2022-01-13

**Authors:** Min Hu, Wen Chen, Winnie Yip

**Affiliations:** 1 School of Public Health, Fudan University, Shanghai, China; 2 TH Chan School of Public Health, Harvard University, Boston, USA

**Keywords:** Hospital management, LMICs, Target, Personnel, Health reform

## Abstract

**Background:**

Although management is important in healthcare, low- and middle-income countries (LMICs) have little experience measuring the competence of hospital management. While improving hospital management is the main focus of hospital reform in China, few studies have empirically documented the baseline situation to inform policy design.

**Methods:**

We assessed the management practices of county-level hospitals in Guizhou in southwest China during 2015. We used the Development World Management Survey (D-WMS) instrument to interview 273 managers in 139 hospitals. We scored the management practices of the sampled hospitals, overall and in four dimensions (operations, monitoring, targets, personnel management) and three processes (implementation, usage, monitoring). We then converted the scores to the WMS scale and compared these with data from two other LMICs and seven high-income countries (HICs).

**Results:**

On a scale of 1 (‘worst practice’) to 5 (‘best practice’), the mean (SD) hospital D-WMS scores were 2.57 (0.46) overall; 2.71 (0.48), 2.64 (0.58), 2.40 (0.64), and 2.56 (0.40) for operation, monitoring, target, and personnel, respectively; and 2.43 (0.48), 2.62 (0.48), and 2.66 (0.47) for implementation, usage, and monitoring, respectively. After conversion to WMS scores, China ranked seventh of 10 countries, after six HICs and higher than one HIC and two other LMICs (Brazil and India). China ranked higher than the two LMICs in each of the four dimensional scores.

**Conclusions:**

Chinese county-level hospitals should improve their low quality of management by prioritizing target-setting and process implementation, particularly in personnel management. Meanwhile, modern management training should be given to most clinical managers.

**Supplementary Information:**

The online version contains supplementary material available at 10.1186/s12913-021-07396-y.

## Introduction

Healthcare systems are under severe pressure due to aging populations, rising costs of medical technologies, budget austerity, and increasing patient expectations. One potential way to tackle these pressures is by improving hospital performance, i.e., efficiency and quality of care, potentially through better management practices [1]. Evidence from high-income countries (HICs) has also shown that better hospital management scores are associated with being a high-quality hospital [2]. Data from low- and middle-income country (LMIC) also show that better hospital-level cardiovascular management practices are associated with better clinical outcomes (for major adverse cardiovascular events) [3]. Thus, it is important to accurately assess and try to improve hospital management practices, as they may greatly impact healthcare outcomes.

China first introduced a hospital accreditation system in 1989. In 2005, the former Ministry of Health issued ‘the hospital management assessment guidelines’, which were revised in 2008 to help hospitals improve their management practices. However, China has limited empirical evidence (using quantitative measures of hospital management) [4–6] that variation in hospital performance is linked to management fundamentals (setting targets, establishing incentives, and monitoring performance) [7].

In recent years, reform of China’s hospitals has been a priority. Reforms have focused on revamping governance at public hospitals, improving management, strengthening performance monitoring, and changing provider payment methods [8]. Private hospitals have also received government guidance and support, and undergone major reforms in the last 10 years, including in management practices. In rural areas, the public hospital reform started with county hospitals, as they made up the largest proportion of rural public hospitals. The improvements were then expected to be passed downwards through the healthcare system to township health centres and village clinics, which need improved managerial expertise [9]. Thus, there is a need to robustly evaluate hospital management practices in rural China, firstly in county-level hospitals, to establish a baseline. The effectiveness of policy interventions can then be ascertained.

The World Management Survey (WMS) tool uses open-ended questions from independent assessors (analysts) who then score organizational management practices [10–13]. The WMS was initially applied to the manufacturing industry but has since been used in > 2000 hospitals in seven HICs and two LMICs [14, 15]. We used an adapted version of the WMS tool—the Development WMS (D-WMS) [16] that was designed for use in developing countries—to measure hospital management in Chinese county-level hospitals. Our results will add knowledge of hospital management measurement in China [4–6, 17, 18] and help us to understand which particular aspects of management are most important to provide insights into how to improve hospital management quality in China. We also converted our scores to WMS-comparable scores for comparison with other countries (seven HICs and two other LMICs).

## Methods

### Study location

We carried out this cross-sectional survey in Guizhou province, a low-income province in southwest China with a large rural population of approximately 18 million in 2020, accounting for 47% of the population [19]. With a per capita annual income of 26,743 CNY in urban areas and 8090 CNY in rural areas (approximately US$4030 and US$1220, respectively), Guizhou was the seventh poorest out of 34 provinces in China in 2016. Although Guizhou was initially identified for this study as a related research project was already underway there, in 2016 Guizhou ranked sixth (median) in GDP of 12 western provinces. Consequently, Guizhou could be considered as being representative of China’s western region. Guizhou province has 88 county-level units across nine municipalities (sub-provincial level). On average, each county in Guizhou has six private general or specialty hospitals, but most private hospitals are small-scale and low-level, so public hospitals dominate service provision.

### Sampling

Our sample drew randomly from 58 counties across eight out of nine municipalities (Additional file 1). In each county, we included all the public non-specialty hospitals, i.e., county general hospitals and county traditional Chinese hospitals. We also sampled the larger private non-specialty hospitals, by including all those that met any of the following conditions (in 2014): secondary hospital; > 150 beds; inpatients accounted for ≥5% of those in the county; medical expenses incurred accounted for ≥5% of the county total.

In each sampled hospital, the survey was completed by one director and one charge nurse from the first available of: orthopaedic, cardiology, or surgical departments. In accordance with WMS methodology [16, 20], middle managers at this level were purposely selected because they were senior enough to have a comprehensive view of management practices but not so senior as to be detached from day-to-day operations. These three departments were selected because they are relatively more likely to be standards-driven, with consistent processes and protocols, than other departments. This is also in line with previous studies [20–22].

### Measurement instrument

**Fig. 1:**
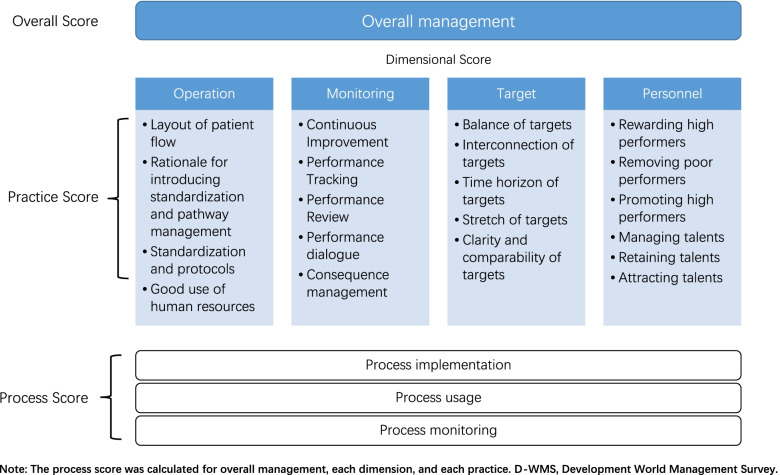
Conceptual model of the D-WMS

In order to measure management practices across hospitals, we drew on an expanded evaluation tool based on the original WMS Questionnaire [11]. The original WMS elicited answers to 20 questions to measure hospital management practices in four broad dimensions: (i) standardizing operations (4 questions); (ii) monitoring performance (5 questions); (iii) setting targets (5 questions); and (iv) incentivizing personnel (6 questions) (Fig. 1). These were evaluated and scored (in whole numbers) from 1 (‘worst practice’) to 5 (‘best practice’) on a pre-defined scoring grid [10]. Total scores were then divided by 20 (for the overall score) or by the number of questions in the dimension to give scores from 1 to 5.

While this scoring system is able to distinguish between well-managed and poorly managed hospitals, scores within LMICs tend to be clustered at the low end of the scale. In order to distinguish between hospitals within these clusters, Lemos and Scur developed the D-WMS, which has an expanded scoring grid that allows for half points but otherwise leaves the 1–5 scale unchanged [16].

In order to compare scores on this D-WMS with scores from other countries that were based on the original WMS, all half points were reduced to the nearest whole number [16]. Scores were always rounded down, as practices that were good enough to achieve the next highest score would have done so [16].

Compared with the original WMS, the D-WMS further divides each management practice into three key processes: (i) process implementation (formulating, adopting, and putting management practices into effect); (ii) process usage (carrying out and using management practices frequently and efficiently); and (iii) process monitoring (monitoring the appropriateness and efficient use of management practices). The D-WMS tool therefore has 60 scores (relative to the original WMS’s 20) to allow for deeper understanding of the drivers of each management practice score [16].

### Interviewers and interview process

To conduct the interviews, we hired and trained a team of 40 public health and health management teachers and students from the two chief medical universities in Guizhou who had medical or management knowledge. These interviewers received extensive training on hospital management, the information they were seeking, and how to probe for further information.

Following the WMS ‘double-blind’ and ‘double-scored’ methodology, each manager was interviewed in person by two interviewers [10, 16]. The interviews were ‘double-blind’ in that managers were not told in advance that they were being scored or shown the scoring grid, while the interviewers were not told in advance about the organization’s performance. ‘Double-scoring’ was achieved by including a second interviewer, whose main role was to monitor the quality of the interview by taking notes and separately scoring the responses after the interview. The two interviewers would then discuss their individual scores to correct for any misinterpretation of responses.

Additionally, we used a variety of procedures to obtain a high success response rate of interview and to remove potential sources of bias from our estimates, including obtaining government endorsements and not asking interviewees for performance or financial data.

### Analysis strategy

Firstly, we calculated the overall and dimensional scores. As each interviewee provided information relating to all three processes for each of the 20 practices, we first took a simple average of these three sub-scores to construct a single score representing each interviewee’s perspective on each of the management practices. As we interviewed two managers at each hospital, we then assigned hospital scores for each practice by averaging the scores given by each pair of interviewees. Based on the hospital score data, we created scores (1–5) for overall management (average of all 20 practices) and dimensional management – operations, monitoring, targets, and personnel management.

We then examined the scores in each process – implementation, usage, and monitoring. To construct these, we omitted the above first step of averaging across the three processes for each practice and reorganized the dataset into three new sets of 20 practices by process. We took the scores for each of the 60 processes and constructed average indices for overall management and dimensional management across every dimension for each process.

To allow us to compare our results with those from nine other countries (seven HICs [US, UK, Sweden, Germany, Canada, Italy, France; all in 2009] and two LMICs [Brazil in 2012 and India in 2013]) that had been assessed with the original WMS using random national samples [23], we converted our D-WMS scores by coding down the half-point scores, e.g., 1.5 in the D-WMS was rounded down to 1 [16]. Our converted scores were compared with scores derived from the original study [23]; while dimension scores for the other nine countries were supplied by the authors of the original study.

## Results

### Sample characteristics

From a list of 146 hospitals (including all 106 public hospitals and 40/379 private hospitals in the eight municipalities), 139 hospitals (response rate 95.2%) in 58 counties in Guizhou were included. Of these, 67.6% were general hospitals and 75.5% were public hospitals (Table 1). Almost all (91.3%) of the hospitals had directors with medical backgrounds, and 50.0% of them had directors with some management training.

**Table 1:** Characteristics of the sample hospitals and interviewees

Sampled hospitals	N	n (%) or mean (SD)
	139	
Hospital type		
General hospital	139	94 (67.6%)
Traditional Chinese hospital	139	45 (32.4%)
Public hospital	139	105 (75.5%)
Number of staff	136	285 (199)
Number of medical staff	137	226 (164)
Number of beds in operation	137	275 (196)
Number of ICU beds	123	3.8 (4.8)
Annual revenue (million CNY [million US$])	136	60.9 (53.1) [9.2 (8.0)]
Annual expenditure (million CNY [million US$])	135	59.2 (51.6) [8.9 (7.8)]
Medical background of director	103	94 (91.3%)
Management training of director	104	52 (50.0%)
**Sampled interviewees**	273	
Age (years)	254	39.4 (8.6)
Male	273	129 (47.3%)
Duration in hospital (years)	266	14.2 (9.5)
Duration in position (years)	252	4.6 (4.3)
Management training	257	33 (12.8%)
Department		
Orthopaedics	273	165 (60.4%)
Surgery	273	64 (23.4%)
Cardiology	273	15 (5.5%)
Other	273	29 (10.6%)
Job title		
Charge nurse	273	136 (49.8%)
Department director	273	127 (46.5%)
Vice director	273	7 (2.6%)
Other	273	3 (1.1%)
Background		
Medicine	260	146 (56.2%)
Nursing	260	106 (40.8%)
Other	260	8 (3.1%)
Education level		
High school or less	259	26 (10.0%)
College	259	85 (32.8%)
Bachelor’s degree	259	136 (52.5%)
Master’s degree	259	10 (3.9%)
Other	259	2 (0.8%)

*SD* Standard deviation

We interviewed 273 managers from the 139 hospitals during July through August 2015, mainly (60.4%) from orthopaedic departments (Table 1). Most of the interviewees were charge nurses (49.8%) or department directors (46.5%); and most (96.9%) had backgrounds in medicine or nursing, but only 12.8% had any formal management training.

### Overall score

**Fig. 2:**
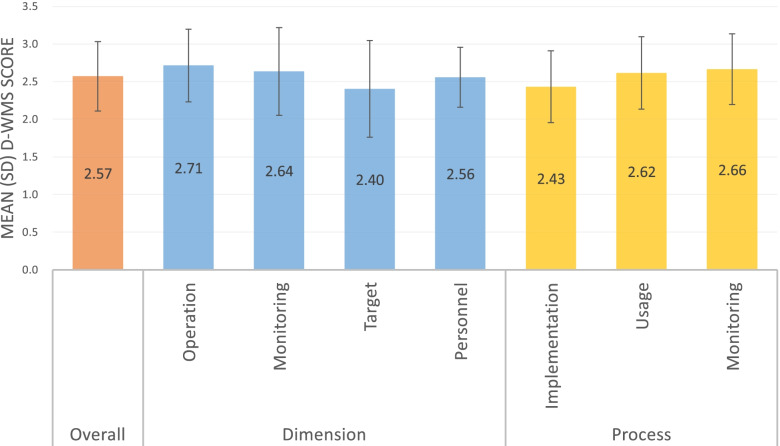
Mean (SD) overall, dimension, and process management scores. *D-WMS* Development World Management Survey; *SD* standard deviation

The mean (standard deviation [SD]) overall management practice (D-WMS score) of the Guizhou sample was 2.57 (0.46) (Fig. 2).

### Dimensional scores

The Guizhou hospitals had their best mean score in operations management (a description of each practice is provided in Table 2). The sampled hospitals performed best in ‘rationale for introducing standardization and pathway management’ (mean score: 2.83) and worst in ‘standardization and protocols’ (2.60) (Table 2). Within performance monitoring, the sampled hospitals performed well in consequence management (3.00), as managers quickly identified and addressed problems, but poorly in performance review, tracking, and dialogue (2.55, 2.50, and 2.42, respectively). The worst target-setting practice score was for clarity and comparability of targets (1.98). It should also be noted that target-setting had the largest variation in scores. Within personnel management, on average, the hospitals were good at managing talent (3.03) but weak in retaining talent (1.85). In fact, retaining talent was the worst of all 20 practices.

**Table 2:** Ranks within dimension and scores of practices

Dimensions and rank within the dimension	Management practice	Description	Management question example	Score, mean (SD); range
Operations-1	Rationale for introducing standardization and pathway management	Tests the motivation/impetus behind changes to operation, what change story was communicated	How often do you challenge/streamline the patient pathway?	2.83 (0.56); 1.00–4.00
Operations-2	Layout of patient flow	Tests how well the patient pathway is configured and improved	Can you briefly describe the patient journey or flow for a typical episode?	2.72 (0.47); 1.50–4.08
Operations-3	Good use of human resources	Tests whether staff are deployed to do what they are best qualified for	How do you know which tasks are best suited to different staff?	2.71 (0.72); 1.00–4.33
Operations-4	Standardization and protocols	Tests if there are standardized procedures that are applied and monitored systematically	How clear are clinical staff members about how specific procedures should be carried out?	2.60 (0.73); 1.00–4.67
Monitoring-1	Consequence management	Tests whether differing levels of performance (NOT personal but plan/process-based) lead to different consequences	How long is it between when a problem is identified to when it is solved? Can you give me a recent example?	3.00 (0.81); 1.00–4.83
Monitoring-2	Continuous improvement	Tests processes for, and attitudes towards, continuous improvement; and whether learning is captured and documented	How do problems typically get exposed and fixed?	2.71 (0.73); 1.00–4.00
Monitoring-3	Performance review	Tests whether performance is reviewed with appropriate frequency and communicated to staff	How do you review your main performance indicators?	2.55 (0.76); 1.00–4.00
Monitoring-4	Performance tracking	Tests whether performance is tracked using meaningful metrics and with appropriate regularity	What kind of performance or quality indicators would you use for performance tracking?	2.50 (0.62); 1.00–4.33
Monitoring-5	Performance dialogue	Tests the quality of review conversations	How are these meetings structured? How is the agenda determined?	2.42 (0.77); 1.00–4.50
Targets-1	Stretch of targets	Tests whether targets are appropriately difficult to achieve	How tough are your targets? How pushed are you by the targets?	2.57 (0.71); 1.00–3.83
Targets-2	Interconnection of targets	Tests whether targets are tied to hospital objectives and how well they cascade down the organization	What is the motivation behind these goals?	2.57 (0.76); 1.00–4.67
Targets-3	Balance of targets metrics	Tests whether targets cover a sufficiently broad set of metrics	What types of targets are set for the hospital? What are the goals for your specialty?	2.46 (0.73); 1.00–4.25
Targets-4	Time horizon of targets	Tests whether the hospital has a ‘3 horizons’ approach to planning and targets	What kind of time scale are you looking at with your targets?	2.45 (0.82); 1.00–4.33
Targets-5	Clarity and comparability of targets	Tests how easily understandable performance measures are and whether performance is openly communicated	Does anyone complain that the targets are too complex?	1.98 (0.65); 1.00–3.42
Personnel-1	Managing talent	Tests what emphasis is put on talent management	How do you ensure you have enough staff/nurses of the right type in the hospital?	3.03 (0.58); 1.67–5.00
Personnel-2	Removing poor performers	Tests whether the hospital can deal with underperformers	How long is under-performance tolerated? How difficult is it to dismiss a nurse/clinician?	2.93 (0.61); 1.17–4.50
Personnel-3	Promoting high performers	Tests whether promotion is performance based	How do you make decisions regarding progression/promotions within the unit/hospital?	2.73 (0.54); 1.08–4.00
Personnel-4	Rewarding high performers	Tests whether good performance is rewarded proportionately	How does your staff’s pay relate to the results of this review? How does the bonus system work?	2.58 (0.70); 1.00–4.17
Personnel-5	Attracting talent	Tests the strength of the employee value proposition	If I were a top nurse/clinician and you wanted to persuade me to work at your hospital, how would you do this?	2.23 (0.46); 1.17–3.17
Personnel-6	Retaining talent	Tests whether the hospital will go out of its way to keep its top talent	If you had a top performing manager, nurse, or clinician who wanted to leave, what would the hospital do?	1.85 (0.39); 1.00–3.33

*SD* Standard deviation

### Process scores

Hospitals in Guizhou generally performed better in process monitoring (mean score: 2.66) and process usage (2.62) than in process implementation (2.43) (Fig. 2).

We also scored the variation across the practices within each dimension. In target-setting, hospitals performed similarly poorly in all three processes (Additional file 2). Within the second-poorest dimension, personnel, hospitals scored lowest in implementation. In fact, the worst implementation performance across all dimensions occurred in the personnel dimension.

### Comparison with other countries

Our D-WMS scores were transformed into internationally comparable WMS scores by rounding down all half-scores. Overall, Guizhou hospitals were ranked seventh out of 10, after six HICs but above France and the other two LMICs, although it should be noted that some of the scores were very similar, so the overall rank order should be interpreted with caution (Table 3).

**Table 3:** International comparisons^a^ of hospital overall and dimensional management scores (comparable)

	Overall	Operations	Monitoring	Targets	Personnel	Generalized operations^**b**^
US	3.00 (1st)	3.03 (1st)	3.21 (1st)	2.87 (1st)	2.92 (1st)	3.04 (1st)
UK	2.69 (2nd)	2.91 (2nd)	2.99 (2nd)	2.55 (3rd)	2.37 (5th)	2.81 (2nd)
Sweden	2.68 (3rd)	2.52 (8th)	2.99 (2nd)	2.75 (2nd)	2.46 (2nd)	2.77 (3rd)
Germany	2.64 (4th)	2.78 (5th)	2.85 (4th)	2.55 (3rd)	2.45 (3rd)	2.72 (4th)
Canada	2.52 (5th)	2.78 (5th)	2.82 (5th)	2.44 (5th)	2.17 (7th)	2.67 (5th)
Italy	2.48 (6th)	2.85 (4th)	2.67 (6th)	2.33 (6th)	2.20 (6th)	2.60 (6th)
Guizhou (China)	2.43 (7th)	2.56 (7th)	2.52 (8th)	2.27 (8th)	2.40 (4th)	2.44 (8th)
France	2.40 (8th)	2.87 (3rd)	2.59 (7th)	2.29 (7th)	2.03 (8th)	2.56 (7th)
Brazil	2.19 (9th)	2.38 (9th)	2.47 (9th)	1.99 (9th)	1.98 (9th)	2.27 (9th)
India	1.90 (10th)	2.11 (10th)	2.03 (10th)	1.55 (10th)	1.93 (10th)	1.88 (10th)

^a^All countries except Guizhou (China) were surveyed and scored using WMS; the Guizhou D-WMS data were converted to comparable scores. Data are derived from the original study [23]; while dimension scores for the nine other countries were supplied by the authors of the original study

^b^Overall excluding personnel

*D-WMS* Development World Management Survey, *UK* United Kingdom, *US* United States, *WMS* World Management Survey

Guizhou surpassed each of the two other LMICs in all four dimensions (Table 3). Although our sampled hospitals scored lower than the average of the seven HICs in operations, monitoring, and target-setting, the personnel dimension scores were similar (Additional file 3). Guizhou scored better in personnel management than France, Italy, Canada, and the UK – the personnel score was Guizhou’s highest-ranked dimension (Table 3). If we exclude the personnel dimension from the overall score, which reflects generalized operations management [16], China was ranked eighth, after France (Table 3). Interestingly, only Guizhou, India, and the US performed better in personnel management than in target-setting.

## Discussion

By interviewing 273 department managers from 139 county-level hospitals (105 public and 34 private) in Guizhou using the D-WMS instrument, to our knowledge, we are the first to use an internationally validated [24] survey tool to measure management practices in county-level hospitals in China. Our study therefore adds data on hospital management practices in a third LMIC, enabling us to identify which management capacities require improvement and to compare our Chinese data to that of other countries.

Previous Chinese studies have used tools based on the WMS, including the WMS-Hospital (WMS-H) Chinese version [17], the Chinese Hospital Management Survey (CHMS) [4, 18], the Development-CHMS (D-CHMS) [5], and the Hospital Management Practice (HMP) rating scale [6, 25]. These included 17 (HMP [6, 25]), 20 (WMS-H [17] and CHMS [4, 18]) or 21 (D-CHMS [5]) practices, which were similar to those included in the WMS—but modified for the Chinese context—across four dimensions. While comparisons between studies should be undertaken with caution, especially given differences in the scoring systems, how the questions were asked, and the study years, scores from the earlier studies [4–6, 17, 18, 25] were generally higher than in the present study. This is likely because the earlier studies included only tertiary hospitals [17] or a mixture of secondary and tertiary hospitals [4, 5, 18]. After accounting for these differences in hospital types, our scores were generally comparable to those from the studies that mainly used Chinese-adapted tools [4–6, 17, 18]. However, we feel that our international tool (D-WMS) is more useful for comparing results to those from other countries as we used a standardized tool that not specifically adapted to Chinese context.

In our international comparison, the sampled county-level hospitals in southwest China ranked seventh overall among 10 countries examined – below all of the HICs except France and above the other two LMICs (Brazil and India). These results show that management practices in rural China need to be improved to reach the standards found in HICs. However, it should be noted that our hospitals had some important differences relative to those in the other countries [23]: they were regional rather than national; they had fewer staff (mean: 285 versus 558 to 2344); and they were lower-level (no teaching hospitals versus 9–42% teaching hospitals) [12]. As previous studies have shown that management scores are positively associated with hospital size [14, 26] and development level [24], this likely overstated the between-country differences.

However, we found that our Chinese hospitals achieved higher scores than those in the other LMICs, overall and in all of the domains. Our results can also be compared to those from a study in Kerala, India [3], which like us, used the D-WMS. Our data showed better management practices in Guizhou than in Kerala, overall and in each of the four dimensions, with the largest difference between Guizhou and Kerala in target-setting [3]. While most of the countries in our international comparison scored worst in personnel management, Guizhou scored worst in target-setting. Target-setting was the key weakness of hospitals in China, though with large variation across hospitals. Another recent Chinese study, which surveyed 95 county hospitals across rural China, has also reported lower scores for target management than for operation, talent, and performance management (39 vs 53–56 on a scale of 0–100) [25]. Poor target management has also been observed in India, in hospitals, retail, and schools [24]. Brazilian hospitals have also performed poorly in target-setting. Those goals stated by our interviewees were rarely set based on internal factors in ways that would enable their use to measure realistic progress from year to year. Disappointingly, quality of care and patient outcomes were generally not priorities. Moreover, hospital goals were typically short- or medium-term rather than long-term, with little prospect of becoming long-term goals. As setting targets is fundamental to management, we suggest that county-level hospitals in China prioritise target-setting. This could involve defining clear and measurable short-, medium-, and long-term clinical, efficiency, financial, and operational goals for individuals and departments. As setting and tracking performance was a part of the Chinese government’s hospital reform, further study addressing the reform’s impacts on target-setting and attainment at hospitals would be valuable.

Our sample scored poorly in ‘retaining talent’, ‘attracting talent’, and ‘rewarding high performers’. As county-level hospitals are crucial in the Chinese healthcare system and talent is critical to organizational performance, we strongly suggest that Chinese hospitals actively improve managerial expertise in attracting, retaining, and rewarding staff. That said, Guizhou ranked fourth in personnel management, behind only the US, Sweden, and Germany. The sampled county-level hospitals’ best scores were for ‘managing talent’ and ‘removing poor performers’. There are currently two talent-recruiting and managing systems in Chinese public hospitals: one is strictly controlled by the local human resources authority, which decides the number and types of staff that the hospitals need; the other is run by hospital directors, who can recruit additional staff if necessary. Hospitals also have more autonomy to dismiss staff within the second system. As the survey did not distinguish between these two systems, it might have overestimated the autonomy and capability to recruit and discharge personnel.

The worst process scores were for ‘implementation’, particularly in personnel management, implying that many advanced management theories/tools were not being used. Interviewees acknowledged that departing staff members’ reasons for leaving were rarely sought and that they lacked criteria to distinguish good performers. This shows that management training must be improved in rural China. We found that most hospital mid-level managers had a clinical background and few had management training. Although having physicians in leadership positions is valuable for hospital performance [20, 27], a mixture of clinical and managerial skills may also have a positive impact on hospital management quality and clinical outcomes [23]. Further, the interplay between management education and policy reform is well-known [28, 29]. Hence, we feel that providing training to physician managers in modern health-care-context-related, operational and general management thinking and techniques could improve hospital management [30]. While monitoring such an impact with a survey such as ours could be labor-intensive and costly, it may help to provide a deeper understanding of hospital management.

Finally, since the outbreak of COVID-19 in late 2019, global healthcare systems have been under great pressure, and hospitals, at the forefront of epidemic prevention and control, have faced as many organizational and management challenges as clinical challenges. Previous studies have found that management factors have an important impact on infection control [31]. Hospital leadership styles and human resource management were associated with a hospital’s infection control performance and infection rate, thereby affecting the overall epidemic prevention and control situation [32]. Therefore, in the context of the post-COVID-19 era, it is important to understand the role of hospital management in the ability to respond to sudden public health crises. Future research should focus on the association between hospital management factors and the effectiveness of COVID-19 prevention and control.

The main limitation of our study is that our sample only included county-level hospitals in western China, so results may not be generalizable to other Chinese regions or other hospital types. However, we believe that our findings are likely representative of many other LMICs, and highlight the need to improve management in such countries. While we included all public hospitals in the region, we only sampled a minority of private hospitals, so our results are likely less applicable to private hospitals. Lastly, although management quality has been correlated with hospital outcomes in other countries [2, 3, 14, 33–35], it would still be beneficial to examine the link between these factors in the Chinese context.

## Conclusions

Using the D-WMS instrument, this study critically measured the management of county-level hospitals in western China and highlights areas of particular importance for improvement. We found that the quality of management was generally low but better than in two other LMICs (India and Brazil). While target-setting was the weakest dimension, implementation was the weakest management process, particularly in personnel management. Modern management training for clinical managers therefore needs to be intensified. Future studies could examine how management practices in Chinese hospitals have improved and whether this has had any impact on patient outcomes.

## Supplementary Information

**Additional file 1.** Selection of county-level administrative divisions in Guizhou.

**Additional file 2.** Process scores in each dimension.

**Additional file 3.** International comparisons on hospital overall and dimensional management scores (comparable).

## Data Availability

The dataset generated during the current study are not publicly available. The study dataset is available from the corresponding author on reasonable request.

## Abbreviations

CHMS: Chinese Hospital Management Survey
D-CHMS: Development Chinese Hospital Management Survey
D-WMS: Development World Management Survey
HIC: High-income countries
HMP: Hospital Management Practice
LMIC: Low- and middle-income country
SD: Standard deviation
WMS: World Management Survey
WMS-H: World Management Survey Hospital.
