# Supplementary material for: Hospital management practices in county-level hospitals in rural China and international comparison
Source: BMC Health Serv Res. 2022 Jan 13;22:64. doi: 10.1186/s12913-021-07396-y (PMC8755900; doi:10.1186/s12913-021-07396-y)
Supplement: Supplementary file 1 — Additional file 1. Selection of county-level administrative divisions in Guizhou. [file 12913_2021_7396_MOESM1_ESM.docx]

**Additional File 1. Selection of county-level administrative divisions in Guizhou**

County-level administrative divisions in Guizhou

N = 88

Divisions of which rural population made up larger than 50% of total population

n = 74

Divisions of which rural population made up less than 50% of total population

n = 14

Divisions included in the national county hospital pilots

n = 8

Divisions in which hospital funding was mainly administered by the Department of Human Resource and Social Security

n = 8

Divisions further included for sample of hospitals

n = 58

Divisions in which hospital funding was mainly administered by the Department of Health

n = 66
