# Supplementary material for: Hospital management practices in county-level hospitals in rural China and international comparison
Source: BMC Health Serv Res. 2022 Jan 13;22:64. doi: 10.1186/s12913-021-07396-y (PMC8755900; doi:10.1186/s12913-021-07396-y)
Supplement: Supplementary file 2 — Additional file 2. Process scores in each dimension. [file 12913_2021_7396_MOESM2_ESM.docx]

**Additional file 1** Process scores in each dimension.

*D-WMS* Development World Management Survey; *SD* standard deviation.

**Additional file 2** International comparisons on hospital overall and dimensional management scores (comparable).


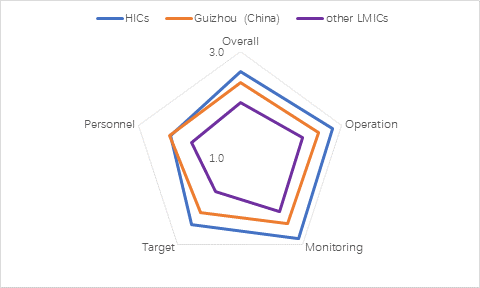


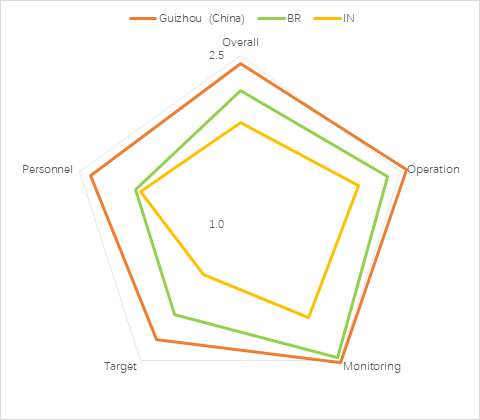


Note: Other samples except Guizhou (China) were surveyed and scored using WMS, while the Guizhou D-WMS data were converted to comparable scores. *BR* Brazil; *D-WMS* Development World Management Survey; *HIC* high-income county; *IN* India; *LMIC* low- and middle-income country; *WMS* World Management Survey.
