# Supplementary material for: Hospital management practices in county-level hospitals in rural China and international comparison
Source: BMC Health Serv Res. 2022 Jan 13;22:64. doi: 10.1186/s12913-021-07396-y (PMC8755900; doi:10.1186/s12913-021-07396-y)
Supplement: Supplementary file 3 — Additional file 3. International comparisons on hospital overall and dimensional management scores (comparable). [file 12913_2021_7396_MOESM3_ESM.pdf]

Additional file 1 Process scores in each dimension.

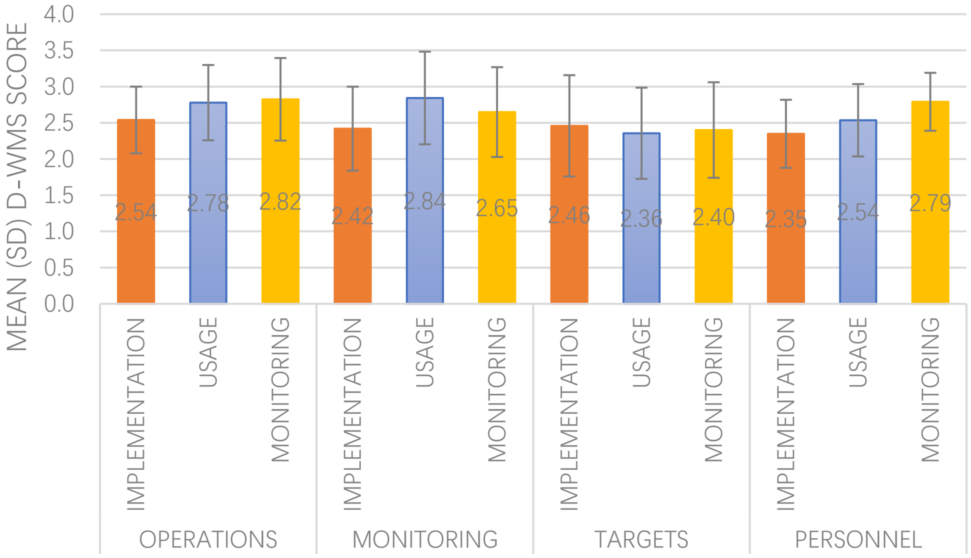

D-WMS Development World Management Survey; *SD* standard deviation.

Additional file 2 International comparisons on hospital overall and dimensional management scores (comparable).

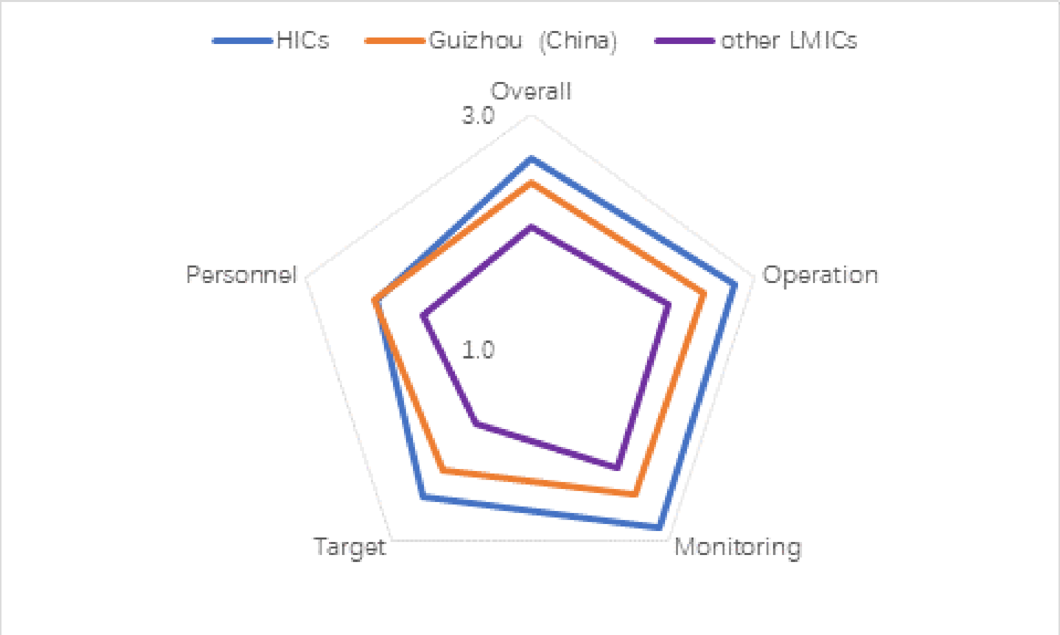

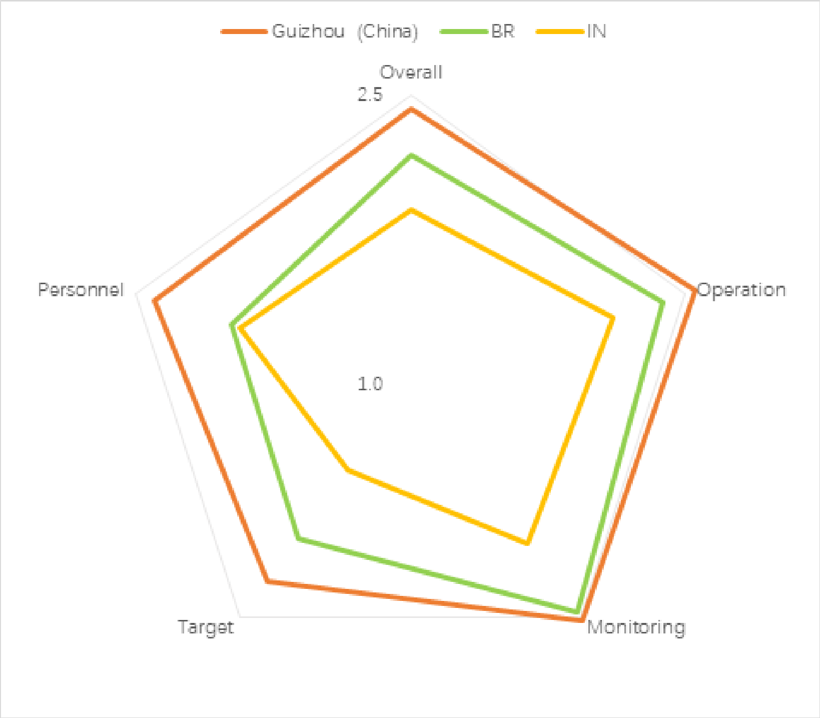

Note: Other samples except Guizhou (China) were surveyed and scored using WMS, while the Guizhou D-WMS data were converted to comparable scores. *BR* Brazil; *D-WMS* Development World Management Survey; *HIC* high-income county; *IN* India; *LMIC* low- and middle-income country; *WMS* World Management Survey.
